# Transthyretin and the brain re-visited: Is neuronal synthesis of transthyretin protective in Alzheimer's disease?

**DOI:** 10.1186/1750-1326-6-79

**Published:** 2011-11-23

**Authors:** Xinyi Li, Joel N Buxbaum

**Affiliations:** 1 Department of Molecular and Experimental Medicine, The Scripps Research Institute, 10550 North Torrey Pines Rd., MEM-230, La Jolla, CA 92037, USA

**Keywords:** Beta-amyloid precursor protein (AβPP, APP), Beta-amyloid (Aβ), Alzheimer's disease (AD), Transthyretin (TTR), Amyloidosis, Protein homeostasis, Aggregation

## Abstract

Since the mid-1990's a trickle of publications from scattered independent laboratories have presented data suggesting that the systemic amyloid precursor transthyretin (TTR) could interact with the amyloidogenic β-amyloid (Aβ) peptide of Alzheimer's disease (AD). The notion that one amyloid precursor could actually inhibit amyloid fibril formation by another seemed quite far-fetched. Further it seemed clear that within the CNS, TTR was only produced in choroid plexus epithelial cells, not in neurons. The most enthusiastic of the authors proclaimed that TTR sequestered Aβ *in vivo *resulting in a lowered TTR level in the cerebrospinal fluid (CSF) of AD patients and that the relationship was salutary. More circumspect investigators merely showed *in vitro *interaction between the two molecules. A single *in vivo *study in *Caenorhabditis elegans *suggested that wild type human TTR could suppress the abnormalities seen when Aβ was expressed in the muscle cells of the worm. Subsequent studies in human Aβ transgenic mice, including those from our laboratory, also suggested that the interaction reduced the Aβ deposition phenotype. We have reviewed the literature analyzing the relationship including recent data examining potential mechanisms that could explain the effect. We have proposed a model which is consistent with most of the published data and current notions of AD pathogenesis and can serve as a hypothesis which can be tested.

## Introduction

All amyloid fibrils are similar in appearance, displaying Congophilic, non-branching fibrils 7.5-10 nm in diameter. The twenty nine (thus far) identified human amyloid precursors [1] share no primary sequence and no common conformation although recent biophysical studies suggest the presence of conformationally/energetically similar repeat subunits which determine whether a given protein belongs to the "amylome" [2]. Further it has been suggested that while the precursors represent a variety of folded and unfolded native structures, a combination of primary structural features and level of expression determines the ordering of proteins along a proposed "edge of stability" under *in vivo *conditions, i.e. there are both qualitative and quantitative factors that influence whether a protein will aggregate *in vivo *[3,4]. The frequency of many of the amyloidoses increases with aging but their deposition appears to be independent, i.e. each has its own anatomically predisposed site and pattern [5]. Thus, while there are reported instances of mixed precursor deposition, they are relatively uncommon, e.g. [6-9]. Nonetheless the commonality of structure that leads precursor proteins to form fibrils suggests that interaction could occur, perhaps accelerating fibril formation. The example of transthyretin (TTR) and β-amyloid (Aβ) raises the question as to whether the effect may be, in truth, to reduce fibrillogenesis.

Wild type and mutant forms of TTR are the precursors in the systemic human diseases, Familial Amyloidotic Polyneuropathy (FAP), Familial Amyloidotic Cardiomyopathy (FAC) and Senile Systemic Amyloidosis (SSA) [10]. In contrast, Alzheimer's disease (AD) is a localized amyloid disease of the brain. AD and the TTR amyloidoses share age dependence and are manifested as both autosomal dominant, mutation-related and sporadic (wild type protein associated) diseases. In the TTR amyloidoses the precursor is synthesized primarily by hepatocytes distant from the main sites of deposition in peripheral nerve and heart. However local synthesis and deposition can be seen in the eye, gut, kidney and choroid plexus. In AD the β-amyloid precursor protein (AβPP) is synthesized ubiquitously but deposition and tissue compromise are restricted to the brain and even more so to specific brain regions.

The first association of TTR with AD was the observation that cerebrospinal fluid (CSF) could inhibit Aβ fibril formation *in vitro *[11]. TTR [12] was the third CSF protein found to interact with Aβ after apolipoprotein E (ApoE) [13] and ApoJ (or clusterin) [14]. It was hypothesized at that time that these three extracellular proteins could "sequester" Aβ, thereby preventing neuronal damage, although there was little evidence presented as to how or where such sequestration could take place. Perhaps "chaperone" in the sense of "protector" might have been a better term than "sequester", but the oxymoronic phrase "pathologic chaperone" had already been utilized to describe the co-deposition of ApoE in AD plaques [15].

Results of the early experiments supporting the association and suggesting that the interaction could be beneficial were suspect because of reservations concerning methodology. Further, the notion that an *in vivo *systemic amyloid precursor could have a salutary effect on the course of another, albeit local, form of amyloidosis, derived from a different precursor, taking place in a different, relatively closed anatomic compartment seemed counterintuitive. Lastly the published evidence that TTR was not a neuronal protein, but synthesized in choroid plexus epithelium made it seem unlikely that it could have much to do with the primarily neuronal degenerative process produced by aggregation of a protein produced in/by neurons [16].

We will review the relevant published papers that have contributed to our current knowledge regarding the relationship between TTR and AD. We will try to point out the inconsistencies that have cast doubt on the pathogenetic importance of the connections and we will present hypotheses that have been proposed to account for the interaction.

### Alzheimer's Disease

The neuropathologic hallmarks of human AD include extracellular senile plaques consisting primarily of fibrils representing aggregated Aβ peptides, intracellular neurofibrillary tangles composed of hyperphosphorylated microtubule-binding tau protein [17-19], and synaptic and neuronal loss particularly in the hippocampus and cortex, the regions associated with cognition and memory (reviewed in [20]). In addition inflammation (reviewed in [21,22]), oxidative damage (reviewed in [23-25]) and reactive gliosis [26] are evident in AD brains.

The precise molecular mechanisms responsible for the pathology of AD are still unclear although there is no lack of reasonable models. Since the original isolation and identification of Aβ and AβPP, the weight of clinical and experimental evidence supports a major, if not primary role for Aβ in the development of AD (reviewed in [27-29]). Whether it is the ultimate source of the pathology is uncertain but the evidence for involvement of AβPP in AD pathogenesis is convincing.

### AβPP processing pathways

**Figure 1:**
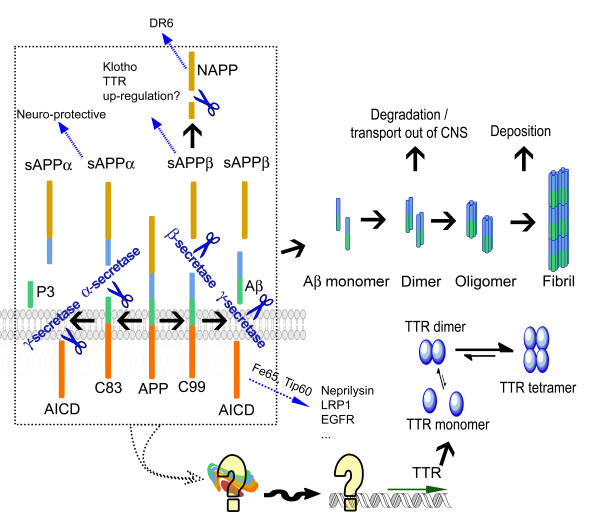
**Amyloid precursor protein (APP) processing pathway **[30-62] . AICD, AβPP intracellular domain; C83 (C99), carboxy-terminal fragments C83 (C99); DR6, death receptor 6; LRP, lipoprotein receptor-related protein; sAPP, secreted AβPP fragment; TTR, transthyretin. Degradation, see 'Clearance of Aβ' of the text for details. Involvement of TTR regulation: it has been suggested that APP or its fragments up-regulate TTR [169,171,230].

AβPP is a 695-770 amino acid glycosylated membrane protein with a single hydrophobic transmembrane domain of 23 residues [30]. A large hydrophilic amino-ectodomain of AβPP is cleaved by α- or β-secretase to produce secreted AβPP fragments, sAPPα or sAPPβ, respectively [31] (see Figure 1). Alpha-secretase is a member of the ADAM (a disintegrin and metalloprotease) family of proteases anchored in the cell membrane [32-36], which includes ADAM9 [33], ADAM10 [35], ADAM17 (also known as tumour necrosis factor-α convertase, TACE) [37] and ADAM19 [36]. The sAPPα fragment appears to be involved in the development of the nervous system, promoting neurite outgrowth [38,39], synaptogenesis [40,41], enhancing memory formation [42], and providing neuro-protection against excitotoxic stimuli [43] and metabolic and oxidative insults [44] (For review see [45]).

Beta-secretase (BACE-1) is a predominantly β-site amyloid precursor protein-cleaving aspartyl-protease [46,47]. However the earlier notion that BACE-1 is the only β-secretase activity protease *in vivo *[48,49] has been challenged [50-52]. The BACE-1 cleavage product, sAPPβ, does not have the same neuroprotective properties as sAPPα. It has been recently shown that upon further cleavage, sAPPβ releases a 35 kDa amino terminal fragment (N-APP), representing amino acids 1-286 of AβPP, that behaves as a ligand for neuronal death receptor 6 (DR6) [53]. That molecule has been hypothesized to be involved in pruning of synapses during development of both central and peripheral neurons [53]. The investigators suggested that aberrant activation of the "death signal" could play a role in AD pathogenesis but there is no published direct evidence for such an effect in AD (Figure 1).

Alpha and β cleavages also yield small cytoplasmic carboxy-terminal fragments (CTF's), CTF83 and CTF99, respectively. In the so-called non-amyloidogenic pathway, CTF83 is digested by γ-secretase, a complex with presenilin 1 at its catalytic core [54] (for γ-secretase reviews see [55-59]), releasing the APP intracellular domain (AICD) which may contribute to the toxicity of AD [60] and the extracellular p3 element with as yet no known function (reviewed in [61,62]). In the amyloidogenic pathway, CTF99 is cleaved by γ-secretase resulting in AICD and small peptides called Aβ ranging from 38 to 43 amino acids. Aβ_1-40 _and Aβ_1-42 _are the dominant forms in senile plaques [63,64]. Aβ_1-42 _is more amyloidogenic and more prevalent in plaques than Aβ_1-40 _[64] but the latter is more abundantly secreted by cultured cells [65,66]. Aβ_1-42 _is generated in the endoplasmic reticulum/Golgi/intermediate compartment (ERGIC) [67,68], while Aβ_1-40 _is generated in trans-Golgi network (TGN) [68]. The endosomal/lysosomal system also plays a role in generating Aβ [69] (Figure 1).

### Clearance of Aβ

The amyloidogenic peptides may be taken up by microglial and astrocytic endocytosis [70,71]; in the brain or by endothelial cells comprising the blood-brain barrier (BBB) [72]. They also form the neuropathologically diagnostic extracellular amorphous or fibrillar deposits (plaques). Some of the released peptides may enter the brain interstitial fluid (ISF) go to the CSF, then to the bloodstream, a pathway which may be responsible for 10%-15% of cerebral clearance of Aβ. The majority of clearance occurs via transport through the BBB [73]. Low-density lipoprotein receptor-related protein (LRP) regulates Aβ clearance by carrying the peptide from brain to blood via transportation across the BBB [73] with the assistance of two other transporter ligands, apoE and α2-macroglobulin (α2M) (reviewed in [74,75]). The receptor for advanced glycation end products (RAGE) is the influx receptor for Aβ [76,77].

Besides taking up soluble and fibrillar Aβ, microglia and astrocytes also secrete proteinases that degrade Aβ extracellularly [71,78]. Aβ can be degraded by a number of proteases including angiotensin converting enzyme (ACE) [79,80], Cathepsin B [81], endothelin converting enzymes (ECE) [82], glutamate carboxypeptidase II [83], matrix metalloproteinases (MMP-2/gelatinase A [84], MMP-9/gelatinase B [85,86]), plasmin [87,88], neprilysin (also known as neutral endopeptidase 24.11 (NEP) and enkephalinase) [89,90] and insulin degrading enzyme (IDE, insulysin) [78,91]. Deficiency of neprilysin [92,93] and IDE [94] caused increased cerebral accumulation of endogenous Aβ in transgenic models of AD *in vivo*. Moreover, overexpression of neprilysin [95,96] and IDE [96] reduced Aβ levels and plaque burden in similar transgenic mice. Lipidated ApoE enhanced degradation of Aβ by neprilysin [97].

### Amyloid hypothesis and alternatives

With Aβ as its focus, the current version of the "amyloid hypothesis" as the etiology of Alzheimer's disease proposes that "soluble oligomers" formed by Aβ are the toxic agents rather than monomers or fibrils. The extracellular oligomers are proposed to induce inflammatory responses, oxidative stress etc. and ultimately cause neuronal spine and synaptic loss through an as yet unknown mechanism [98-101].

There is abundant evidence favoring an Aβ-centric hypothesis. Patients with Down's syndrome, caused by trisomy 21, thus carrying a third copy of the AβPP gene, uniformly develop AD-like pathology after age 40. The increased AβPP gene dose results in elevated Aβ level and early deposition of extracellular Aβ, neuritic plaques and neurofibrillary tangles [102-104]. In familial AD (FAD) mutations of presenilin 1 and 2 and AβPP genes cause early onset FAD with increased amounts of Aβ [105-108] or ratio of Aβ_1-42_/Aβ_1-40 _[109,110]. In an early onset form of AD, the so-called Swedish double mutation (K670N/M671L), cleavage by β-secretase is enhanced with subsequent increased production of total Aβ [111].

Transgenic mouse AD models have been created using genes encoding mutant forms of presenilin and AβPP that have been identified in autosomal dominant forms of human AD. To some extent they all reproduce AD phenotypes, more closely resembling the early stages of the human disease than the globally symptomatic condition (see reviews [112-114]). The AβPP models seem to require multiple copies of the mutant gene, creating a molecular environment more analogous to that in Down's syndrome than in sporadic human AD. It is not certain that the organismal response to multiple copies of a gene encoding a mutant protein is absolutely analogous to the disease produced by an aggregated fragment from two copies of a normal AβPP gene. Nonetheless the molecular events and the pathologic sequellae are similar.

In human AD patients, the severity of pathology correlates best with the concentration of soluble Aβ in the brain, not with that of the insoluble plaques, the morphologic hallmark of AD [115,116]. In brain-slices, dimers and trimers of Aβ are synaptic toxins and oligomers inhibit long term potentiation [117-120]. Recently, it has been suggested that oligomeric Aβ_1-42 _binds to PrP^c ^and inhibits synaptic plasticity [121], however that observation has not been confirmed in all laboratories [122].

Other functional studies have indicated that the degree of dementia in AD is more highly correlated with the presence of neurofibrillary tangles than amyloid plaques [123,124]. However those studies antedated the analyses of soluble Aβ. Patients with mild cognitive impairment (MCI) who develop AD have lower levels of Aβ_1-42_, higher total tau (T-tau) protein, and tau phosphorylated at threonine 181 (P-tau_181_) in CSF than those who do not [125,126]. In CSF of AD patients, the decreased Aβ_1-42 _and increased tau levels appear to be good biomarkers for some purposes [127-129].

In cell culture, *in vitro *synthesized Aβ oligomers are toxic to a variety of cells [130]. When added to primary cultured murine neurons, the oligomers cause synaptic loss, calcium imbalance [131], disruption of mitochondria [132], with subsequent oxidative stress similar to that seen in brains of AD patients [133]. However most *in vitro *toxicity studies used synthetic Aβ peptides and required higher (μM) concentrations than those likely to be encountered *in vivo*. In addition the aggregation-prone nature of Aβ has made it difficult to identify the precise conformations of the toxic species. Despite these correlations, the relationship between Aβ cytotoxicity in tissue culture and the mechanism of neuronal loss in AD is still uncertain.

Hypotheses alternative to the amyloid cascade include a primary effect of ApoE on metabolism [134], membrane dysfunction caused by Aβ dimers [135], primary axonal transport dysfunction [136,137], oxidative stress related to aging, primary mitochondrial dysfunction or cerebrovascular disease [24] and a primary presenilin defect. These challenges to the amyloid cascade hypothesis have persisted (see reviews [138-142]), particularly since clinical trials of agents which targeted clearance of amyloid plaques or inhibition of γ-secretase have failed [143]. Nonetheless the hypothesis remains dominant with the failures being interpreted as being related to either inadequate specificity (in the case of γ-secretase inhibitors, resulting in off-target toxicity), unresponsive stage of disease, or neurovascular inflammation (as with the anti-Aβ antibodies) [144].

### Transthyretin (TTR)

Unlike the circumstance in AD where AβPP is produced and processed in neurons and Aβ aggregates form primarily in the CNS, TTR, a 55 kDa homotetrameric protein, causes disease by depositing as aggregates primarily at a distance from the major site of synthesis. The circulating protein is produced predominantly in the liver, which rarely displays evidence of aggregation or dysfunction. The TTR amyloidoses are prototypical systemic gain of toxic function disorders. The toxic species is comprised of aggregates formed from monomers which misfold after they dissociate from the homotetramer [145]. The most common form of TTR aggregation disease is senile systemic amyloidosis (SSA), caused by wild type TTR protein deposits in the heart, which increases in prevalence in the aged, with frequencies as high as 10-20% in the 9^th ^and 10^th ^decades, perhaps even higher in older groups [146,147]. Mutant TTR protein deposits in peripheral and autonomic nerves and heart are responsible for disease in familial amyloidotic polyneuropathy (FAP), and familial amyloidotic cardiomyopathy (FAC). More than 80 mutants have been reported to be responsible for autosomal dominant deposition disease [148]. CNS deposition has not been noted in FAP except in the choroid plexus and leptomeninges with rare unstable mutants (TTR's D18G, A25T, L12P) and in some cases of individuals carrying more common mutations, e.g. TTR V30M, which are primary sites of TTR synthesis [10]. The carriers of those mutations have a characteristic clinical CNS presentation, even though there does not appear to be actual neuronal involvement by the aggregates [149,150].

TTR is a thyroid hormone (thyroxine (T4)) carrier and the only known plasma retinol (vitamin A) transporter, binding to retinol binding protein (RBP) charged with retinol. The binding sites for its normal ligands, T4 and RBP have been well defined [151-153]. Surprisingly, mice with their endogenous *ttr *gene silenced have no apparent functional phenotype with respect to either thyroid function or vitamin A metabolism as long as vitamin A is supplied in the diet [154-156]. They have been shown to have a behavioral abnormality, the nature of which is currently under active investigation [157,158] and have been reported by one laboratory to have a neuropeptide Y phenotype with obesity and hyperphagia [159]. They also appear to have a defect in peripheral nerve repair in response to injury and an abnormality in the proportion of proliferating to apoptotic cells in the supraventricular zone in the embryonic brain [160,161].

In clinical situations in humans the serum TTR level (0.08-0.45 mg/ml) is used as a marker for malnutrition as well as inflammation, decreasing in both clinical settings [162]. Its serum level is decreased in patients with some tumors [163,164], although it is not clear whether the decrease is related to an inflammatory response or is an intrinsic property of the tumors.

TTR is encoded by a single copy of gene located on chromosome 18 in human and chromosome 4 in mouse. The gene contains four exons with the first exon encoding the leader sequence (reviewed in [10]). The gene is expressed in liver, kidney, pancreas, choroid plexus [165], retinal epithelium, leptomeningeal epithelium [166]. Despite the frequency of cardiac TTR deposition there does not appear to be either TTR gene transcription or TTR protein synthesis in the heart (Buxbaum unpublished). Several groups have shown material reactive with anti-TTR antibodies present in brain parenchyma indicating that the *TTR *mRNA is effectively translated [167-169]. However those observations could also be explained by neuronal endocytosis of TTR synthesized in choroid plexus epithelium [170,171]. More recent studies, using microarray analysis of RNA from carefully dissected regions of brains from multiple animals showed strain and regional variation in *ttr *transcription in different areas of the brain parenchyma [172]. These results differ from those seen in earlier work, using primarily Northern blotting, which suggested that the choroid plexus was the only site of *ttr *transcription in the brain and that the apparent neuronal expression was a function of contamination with choroid plexus or leptomeningeal epithelium [16,173-175]. The most recent results suggest that normally there is a very low level of neuronal TTR synthesis [169,176] with substantial increase in particular pathologic states [169].

### Transthyretin in human AD

Early immunopathologic studies, based on the premise that TTR might be the amyloid precursor in AD, gave conflicting results with respect to the presence of TTR in plaques in human AD brains [177,178]. More recent analyses found TTR co-localized in hippocampal plaques and vessels of AD patients [167,168]. Anti-TTR serum stained the majority of neuronal bodies in AD brains but only 10% of neurons in age-matched non-demented controls [169]. The latter finding may be responsible for the many reports of TTR synthesis only in the choroid plexus in the normal brain since the TTR signal from the choroid plexus, ependyma and leptomeninges is much stronger than that from normal neurons [16,179].

TTR, ApoE and ApoJ (clusterin) are major Aβ-binding proteins in human CSF [12-14]. The mean CSF TTR level has been reported to be decreased in several series of AD patients [180-184]. However not all investigators have found this to be true [185]. The significance of the decrease is not clear. It has been proposed, on the basis of the decrease, that TTR sequesters Aβ but no site of sequestration has been identified. It is also possible that the CSF TTR concentration may be determined in part by neuronal TTR synthesis [169], particularly in AD (as well as choroid plexus production) and that the observed reduction is related to neuronal loss. Also plausible is the possibility that patients with AD have a genetic or acquired low CSF TTR level independent of Aβ binding, which conceivably could put them at greater risk for AD. A recent analysis of *TTR *single nucleotide polymorphisms (SNPs) in the MIRAGE study of AD families has associated 5 *TTR *SNPs with hippocampal atrophy [186]. A prior single small study did not identify AD in carriers of amyloidogenic TTR mutations, but there is no *a priori *reason why such mutations would predispose to Aβ deposition [187].

Reduced CSF TTR levels have also been reported in patients with depression (although not in those who committed suicide), normal pressure hydrocephalus and most recently in amyotrophic lateral sclerosis (ALS) [188-190]. The variability of the finding has made it an unsuitable CSF marker for AD and made it more difficult to understand its role in AD pathogenesis.

An *in vivo *interaction between Aβ/TTR was also noted in human kidneys [191], and in the muscle of a single patient with inclusion body myositis [192]. However its significance in these circumstances is unclear since the subjects did not have clinical AD.

It was hypothesized that TTR could inhibit Aβ related toxicity by sequestration of Aβ thus preventing Aβ aggregation and fibril formation based on the observation that first identified TTR as an Aβ-binding protein in CSF [167]. In subsequent studies of the capacity of a series of recombinant mutant TTR's to inhibit Aβ fibril formation at neutral pH *in vitro *was analyzed. The investigators found that the amount of Congo red binding material formed over a 24-36 hour period was reduced in the presence of many of the recombinant TTR's (at a 5:1 molar ratio of Aβ:TTR). However, the experiments suffered from the lack of non-TTR e.g. albumin, controls and the use of a relative measure of inhibitory capacity that was never quantified in terms of protein concentration. In addition the nature of the Aβ_1-42 _when it was added to the assay was not precisely defined. Given current knowledge regarding the propensity of Aβ to aggregate on standing, it is not clear from the publications whether the different TTR's were actually seeing the same Aβ conformers. Nonetheless in retrospect the observation that TTR bound Aβ and inhibited fibril formation was correct, although the detailed results regarding the relative capacities of different variants are less likely to be valid. The hypothesis itself was attractive since TTR is abundant in Human CSF (5-20 μg/ml or 0.1-0.36 μM) and serum (174-420 μg/ml or 3-7 μM) [12,193]; while Aβ concentration in CSF is relatively low (3 nM or less) [194,195]. However it inferred, as a second hypothesis, that the interaction would be responsible for lowering the CSF TTR concentration.

### Transthyretin in AD worm and mouse models

The initial *in vitro *studies were followed by the intriguing report that in *C. elegans*, wild type human TTR co-expressed with Aβ in body wall muscle cells under control of the same (*unc *54) promoter rescued a phenotype of defective locomotion seen in animals expressing only Aβ [196]. The significance of those data was not clear since no follow up studies were reported in the same system. However with the development of transgenic mouse models of AD it became possible to examine the phenomenon in a more disease-relevant experimental system.

In the transgenic mouse AD model Tg2576 in which the human APP Swedish mutation is expressed under the control of a hamster prion promoter and is associated with plaques, dystrophic neurites, vascular involvement and gliosis, analyses of transcription showed up-regulation of *ttr*. TTR protein was immunochemically detected in neurons in the hippocampus and cerebral cortex, although neuronal-specific *ttr *transcripts were not assessed [171,197]. TTR immunoreactivity was seen in the same areas as the Aβ-staining plaques. Furthermore, injection of anti-TTR antibodies into one ventricle increased Aβ deposition on the injected side relative to that seen in the contralateral cerebral hemisphere [168], suggesting that the reduction of functionally available TTR caused the increased AD-like pathology.

In later studies in the APP23 mouse model (Swedish mutation controlled by the Thy 1 promoter), hippocampal and cortical regions of brains from 15-month old mice showed neuronal staining for TTR and co-staining for Aβ and TTR in the deposits. The blood vessels were also Aβ and TTR positive [158]. Crossing the APP23 mice with a mouse strain over-expressing wild type human TTR under the control of its own promoter (APP23/h*TTR^+^*) normalized cognitive function and spatial learning as well as diminishing the neuropathologic changes and the amounts of Aβ deposited in the animals bearing both constructs [158]. Moreover, APP23/m*ttr*^-/- ^animals showed Aβ deposition in the hippocampus and/or cortex 3 months earlier than in the presence of the *ttr *gene [158]. In animals sacrificed at 5.5 months of age the frequency and amount of Aβ staining and extractable Aβ in the brains of the APP23/m*ttr*^-/- ^were greater than in the APP23/m*ttr*^+/+ ^mice [158]. Another AD transgenic mouse model, the *ceAPPswe/PS1*ΔE9 mouse, hemizygous for a silenced *ttr *allele, also showed earlier deposition than controls but not as early as in the homozygous knockouts in the APP23 mice [198], suggesting a gene dose effect.

Results of experiments examining the effects of silencing the *ttr *gene on other models of AD have not been uniform. In contrast to the results suggesting a salutary effect of TTR in the Tg2576 AD model [171], other investigators reported that total and vascular Aβ burdens in pooled 13-20 month-old Tg2576/*TTR*^-/- ^mouse brains were significantly increased compared to Tg2576/*TTR*^+/- ^mice [199]. The investigators saw no difference in the age of onset and progression between the two strains of mice. However those conclusions were based on examining only two mice from each group per month, which is probably not sufficient to be certain of the observations regarding the pace of development of disease reported in the APP23 mice. In addition homozygous Tg2576/*TTR*^+/+ ^control mice were not included in the study so there was no comparison between *ttr*^+/+ ^and *ttr*^-/- ^animals. Similar studies were performed in TgCRND8 mice, a more aggressive AD model of Aβ deposition in which plaques develop as early as 3 months. The magnitude of spatial memory deficits and Aβ plaque burden were not different in the hippocampi of 6-month-old TgCRND8/*TTR*^+/-^, TgCRND8/*TTR*^-/- ^and TgCRND8/*TTR*^+/+ ^mice [200]. In that model it might have been too late at 4 and 6 months of age to observe significant changes in the rate of development of disease due to the deletion of the *ttr *gene as suggested by the APP23 and *ceAPPswe/PS1*/ΔE9 experiments.

The variability in the results of the gene silencing experiments may be due to differences in the mouse strains studied. Alternatively, since it is clear that mice with two intact copies of the *ttr *gene still develop AD-like pathology, and there is considerable variation in the degree of pathology and behavioral abnormality seen from mouse to mouse in the same strain, it is difficult to get significant results without using relatively large numbers of animals of the same gender, precisely matched for age if one is trying to determine the pace of development of disease, rather than degree of pathology at the endpoint, which may be independent of the presence or absence of TTR. It is possible that examining the knockouts is observing the loss of a physiologic inhibitor/modulator of the pathogenetic process and more subject to mouse to mouse variation, while the over-expressed wild type h*TTR *transgene experiment is more analogous to a pharmacologic manipulation in which the agent is provided in sufficient quantities to overcome individual host differences. It might be useful to cross the h*TTR *over-expressing mice with animals bearing other AD mutants to be certain that the results seen in the APP23 strain were not peculiar to that strain combination.

### Transthyretin and Aβ *in vitro *interaction

In contrast to the results from *in vivo *mouse studies, results from *in vitro *experiments analyzing the interaction between TTR and Aβ are more consistent, particularly in recent years when we have come to understand how to control the behavior of pro-amyloidogenic proteins in solution *in vitro *[201].

Schwarzman *et al *studied 47 recombinant TTR variants (see above). Most (except G42 and P55) bound to Aβ and inhibited Aβ aggregation *in vitro *[202]. But the interpretation of those experiments is subject to some reservations with respect to the experimental methodology (*vide supra*). Wild type human TTR binds to all forms of soluble Aβ, monomer, oligomer and fibrils [158,203-205]. TTR binds to Aβ better at 37°C than 25°C [158], binds to Aβ aggregates better than Aβ monomer [158,205,206], and Aβ_1-42 _better than Aβ_1-40 _[158]. The binding is highly dependent on the quarternary structure of TTR [206]. It has been suggested that human monomeric TTR binds Aβ better than the TTR tetramer. On the basis of tandem mass spectrometry analysis of a glutaraldehyde cross-linked TTR-Aβ fragment, the Aβ binding site appeared to be located in the A strand, in the inner β-sheet and EF helix of TTR [206]. These putative sites must be confirmed independently, using a different methodology. They do not correspond or encompass the sites proposed earlier based on structural modeling [12]. If the sites are correct then mutations in the potential binding residues should reduce affinity or abrogate binding completely.

The stoichiometry and the binding affinity of the Aβ-TTR interaction have been difficult to establish, perhaps because of the tendency of Aβ to aggregate, so that its molecular mass at any moment of the interaction is probably heterogeneous. Using a tryptophan fluorescence quenching method, K_s _was estimated at 2300 M^-1 ^for TTR and Aβ soluble species [205]. The authors noted that the K_s _could be underestimated by several orders of magnitude because the Aβ monomer molecular weight was used in the estimation and it is likely that much of the bound Aβ was heterogeneously oligomeric. Using a competition binding method with radio-iodinated Aβ_1-42 _(presumably a stronger binder than Aβ_1-40_) as the ligand the Kd was estimated to be 28 nM [203]. However there is still lack of independent confirmation of this relatively strong interaction constant. In contrast to other laboratories the same investigators concluded that the binding between TTR and various Aβ species is similar.

Nonetheless, it is now apparent, as originally suggested by Goldgaber and his colleague [12,202], that the interaction between TTR and Aβ interferes with Aβ aggregation *in vitro*. Using a variety of methods at least four other laboratories have now shown that TTR inhibits Aβ fibril formation [158,203-205]. While it appears from some assays that TTR inhibits oligomer formation in others the mechanism of inhibition of fibril formation may be mediated via suppression of large aggregate formation (Li and Buxbaum, unpublished). Two groups have clearly shown that monomeric TTR suppresses Aβ fibril formation better than TTR tetramer (Li and Buxbaum, unpublished) [206]. The interaction between TTR and Aβ species is apparently beneficial to cultured cells under Aβ stress. TTR prevented accumulation of the Aβ In cultured vascular smooth muscle cells [207]. In the human neuroblastoma cell line SK-N-BE, TTR inhibited ultrastructural changes characteristic of apoptosis [204]. Pre-incubation of Aβ with TTR also suppressed caspase-3 activation in the undifferentiated human neuroblastoma SH-SY5Y cell line [203] and the cytotoxicity induced by Aβ oligomers on SH-SY5Y cells differentiated by retinoic acid treatment [169]. Moreover, TTR also inhibited cytotoxicity and the induction of reactive oxygen species (ROS) by Aβ species in cultured embryonic mouse neurons [169].

### Is the beneficial effect of transthyretin direct?

**Figure 2:**
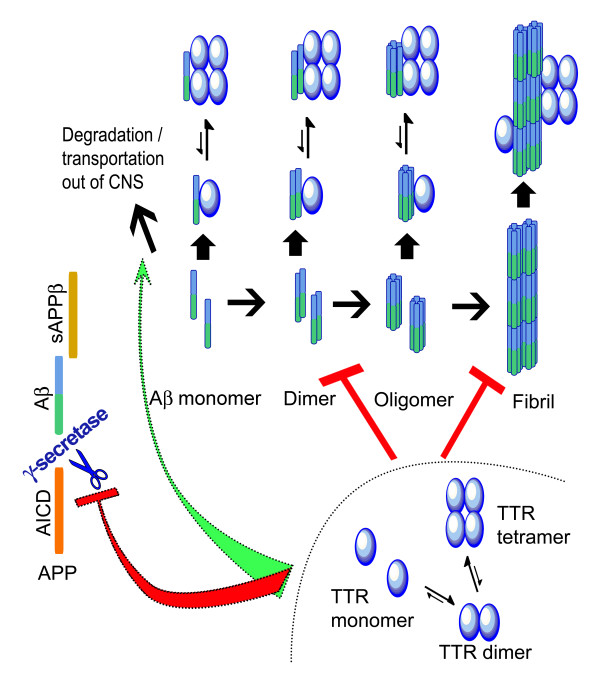
**Proposed mechanisms of TTR inhibition of Ab toxicity**. TTR inhibition of Aβ aggregation (fibril formation) was reported by many groups [12,158,169,203-205] and current evidence suggested that the binding is mediated by association of monomeric TTR to Aβ. It is also possible that TTR facilitates Aβ degradation directly [208] or indirectly, transports of Aβ from CNS into serum (plasma sink hypothesis) [12,202]. TTR may also inhibit Aβ production by inhibition of γ-secretase cleavage [169].

While TTR binding to Aβ appears to be well documented it is not clear how such binding impacts on AD *in vivo*. The notion of "sequestration" has been floated from the very beginning however, where or how the Aβ is being "sequestered" is not apparent (see Figure 2 for hypothesis). It has also been suggested that TTR is a "cryptic protease" and cleaves Aβ [208], with subsequent disaggregation of the fibrils [203]. The data supporting this hypothesis have not been confirmed by other laboratories, either with respect to disaggregation or proteolysis under physiologic conditions. The concentration of recombinant TTR (13.6 μM) used to show cleavage of Aβ [208] is almost twice the level found in the serum and 30 times higher than the concentration of TTR in CSF [12,193]. Since the concentration of TTR in the brain has not been determined these results have to be interpreted carefully.

If the soluble Aβ oligomers or fibrils are the neuro-toxic elements in AD patients or mouse models, the protective property of TTR can be a simple function of inhibiting aggregation and fibril formation by binding Aβ aggregates thus reducing their toxicity, as has been shown *in vitro*. TTR-Aβ complexes have been co-immunoprecipitated from the cerebral cortices of APP23 mice as well as some human AD brains using anti-TTR sera, although the precise conformer of the bound Aβ has not been established [169] (Figure 2).

In the APP23 AD mice over-expressing human TTR the amounts of SDS and formic acid extractable Aβ species were markedly reduced [158]. Thus, in this model the suggestion that given its amyloidogenic property, TTR may bind to Aβ and form large insoluble aggregates, thus protecting neurons from the toxicity induced by soluble oligomeric Aβ species has no experimental support. It is also possible that TTR may bind to AβPP as well inhibiting the cleavage of the AβPP by blocking at or close to α-, β-, or γ-secretase docking sites, thus reducing the amount of Aβ either by facilitating the non-amyloidogenic pathway (by helping α- secretase docking) or by inhibiting the amyloidogenic pathway (preventing β- or γ-secretase docking) [169]. This has been proposed to account for the salutary effect of the Bri2 transgene in a transgenic AD model [209]. It is also possible that TTR binds to the secretases or both the secretases and AβPP (fragments/full-length) and blocks Aβ production. This is another possible explanation for the markedly decreased Aβ_1-40/1-42 _content in the presence of AβPP in the cortex of the h*TTR *over-expressing APP23 mice [169] (Figure 2).

Since the evidence indicates that TTR can bind many forms of Aβ it is also possible that TTR exerts its salutary effect on AD, particularly when it is over-expressed in the APP23 mice, by enhancing the hypothesized "plasma sink" by which Aβ-binding molecules in the circulation shift the equilibrium of newly generated Aβ from the brain, where the aggregates may be cytotoxic to the peripheral circulation, where they can be degraded. This has been proposed as an explanation for the effects of anti-Aβ antibodies, gelsolin and the ganglioside GM1 [210,211]. If that is the case it should be possible to isolate TTR-Aβ complexes from the serum of the human TTR over-expressing APP23 mice.

### Is the beneficial effect of transthyretin indirect?

Despite the evidence supporting a direct interaction between TTR and Aβ-related peptides, the TTR effect might be indirect. As an amyloidosis precursor, TTR could activate the unfolded protein (UPR), and other proteostatic responses thus inducing chaperone transcription, or activating stress related pathways, thus changing the protein homeostasis network to be more efficient in coping with Aβ aggregation [212]. If this is the case, one would expect to find benefits from comparable over-expression of other amyloid precursor proteins. Wild type cystatin C and Bri2, other proteins in which mutations produce CNS amyloid deposition in humans, also inhibit Aβ fibril formation [213-216] (Table 1). Gelsolin, another human amyloid precursor binds Aβ [217] and ameliorates a transgenic AD model even when only expressed peripherally, a phenomenon more likely to reflect a "plasma sink" effect [218]. However each of these proteins binds Aβ directly and their effects cannot be attributed exclusively to stimulation of protein homeostatic mechanisms.

**Table 1:** Do Amyloid precursors "chaperone" Aβ?

Protein	Transgene suppresses	Knock-out accelerates	Cytotoxic inhibition	*In vitro *interaction	In Human AD brains
Transthyretin	+	+	+	+	+
Bri2	+	N.D.	N.D.	+	+
Cystatin C	+	N.D.	N.D.	+	+
Gelsolin	+*	N.D.	N.D.	+	+
Neuroserpin	+**	N.D.	+	+	+

N.D., Not Determined.

*Gelsolin only expressed in livers of transgenic animals.

**Neuroserpin experiments have only been done in transgenic drosophila.

Others have argued that the TTR effect in AD models depends upon its function as an RBP binding protein. Increased TTR could increase the amount of available retinoic acid, thus enhancing neuronal maintenance. Similarly the accelerating effect of the TTR knockout could depend on a relative lack of retinoids in the CNS which amplifies the toxic effect of Aβ. Retinoic acid inhibitors have been shown to compromise neuronal function in older rodents and retinoic acid has been found to enhance performance [219,220]. Thus it would be useful to determine if expressing a human AD gene on an RBP knockout background in the presence and absence of TTR would reveal a different phenotype from that seen when the APP23 construct is expressed in the absence of TTR alone. There are also suggestions that TTR may also be involved in AD through a vascular mechanism. In such a scenario TTR would cleave apolipoprotein A-I (ApoA-I), a constituent of HDL resulting in reduced cholesterol efflux and increased formation of amyloid fibrils [221].

Given the multiple functions of TTR (reviewed in [222]), it also possible that TTR enhances mechanisms that specifically degrade Aβ, or that it plays a currently unknown role in the maintenance of critical neuronal functions.

### Do Alzheimer's peptides regulate neuronal transthyretin expression?

If TTR expression plays an important role in neuronal protection from Aβ aggregation or processing or in the normal function of AβPP, it would seem appropriate for its expression to be regulated by the system involved in the generation of Aβ or its related peptides. In hippocampal slices from Tg2576 AD mice TTR mRNA and protein were increased compared to WT mice [158,168,171]. The same was true in isolated cortex and hippocampus of the APP23 mice. We can also infer that the same is true in human AD since there is little neuronal staining for TTR in non-demented human brains and extensive staining brains from AD patients as reported anecdotally by Goldgaber and Johnson and systematically examined in our laboratory (see above). Since primary cultured neurons derived from 14-16d embryonic mice of the same genotypes show markedly increased expression of the TTR gene, it is safe to say that the increased staining is due to increased synthesis rather than uptake of choroid plexus synthesized TTR [169].

It had previously been suggested that sAPPα might increase TTR transcription, although at that time TTR mRNA had not been demonstrated in neurons [223]. In more recent studies it has been reported that the AICD fragment regulates transcription of other genes through activating Fe65 and the chromatin-remodeling factor Tip60 [224-227]. The genes regulated by AICD include neprilysin, the neutral endopeptidase with Aβ-degrading activity (*vide supra*) [225]; lipoprotein receptor *LRP1 *which is related to cholesterol metabolism and Aβ transport [228]; EGF receptor, whose promoter is bound by AICD and negatively regulated [229] etc. Most recently it has been suggested that the *TTR *and *Klotho *genes are specific downstream targets of sAPPβ [230]. *TTR *and *Klotho *expression are decreased in loss-of-function states but increased in gain-of-function states using transcriptional profiling [230] (Figure 1).

When mice are exposed to environmental "enrichment", both the steady-state levels of Aβ peptides and Aβ deposition in brains of APPswe/PS1ΔE9 are significantly reduced, and *ttr *is one of the genes up-regulated [231]. Similarly administration of *Gingko biloba *and a number of unsaturated fatty acids to rodents have been reported to increase TTR mRNA abundance in cortical neurons as measured by microarray analysis [232-234]. Some of these compounds have had favorable effects in transgenic models of human AD [235,236]. However large studies of at least one of these in human AD patients have failed to show any benefit [237]. Perhaps this is just an example of "too little too late" rather than a conceptual error.

### Summary: Transthyretin, aging and Alzheimer's disease

Over-expression of human TTR suppresses the AD phenotype in a well validated model of human AD. Silencing the endogenous *ttr *gene appears to accelerate the disease but those results are less consistent. The majority of cortical and hippocampal neurons in human AD brains contain TTR protein as do the neurons in several murine AD models. The increased neuronal TTR is the result of increased transcription. *In vitro *interaction between recombinant TTR and synthetic Aβ has been demonstrated in multiple laboratories with the interaction reducing both fibril formation and Aβ-induced cytotoxicity in tissue culture. The interaction has now been shown to occur *in vivo *in both a murine model and in some human AD brains. Further it appears that TTR transcription may be directly influenced by the Aβ precursor. Thus wherever Aβ peptides are produced, i.e. intracellularly in neurons or secreted into the cerebral interstitial space, TTR is available, either on the basis of neuronal (intracellular) or choroid plexus production and secretion. If, as suggested by the *in vitro *studies, the TTR monomer is critical for binding intracellular Aβ, it is likely that newly synthesized peptide rather than dissociated tetramer is the source. Hence we would expect to find Aβ _1-40/42 _and TTR monomer in the same cellular compartment.

In the face of these data suggesting a role for TTR in suppressing the molecular events responsible for clinical AD, one must conclude that with time the amount of pathogenic Aβ peptide production exceeds the neuron's capacity to neutralize it. This neutralizing capacity may be represented by the conventional protein homeostasis network (including the unfolded protein response, heat shock induced chaperones and their co-chaperones, the proteasome ubiquitin system and autophagic responses) [212]. It now appears that in this setting TTR may also comprise part of that network. There are considerable data suggesting that these mechanisms decline with aging. A relative deficiency of any of them may render the normal processes that deal with Aβ or its aggregates unable to compensate for a constant (or increased) aggregate load thus initiating disease. Such a scenario could certainly account for the findings in the over-production models of AD, whether it also applies in sporadic disease is a subject of speculation and further investigation. For the moment any such studies cannot ignore the role of TTR since hippocampal and cortical neurons from human AD and mouse AD model brains seem to increase its production.

### Epilogue: Is transthyretin the only one?

Aβ amyloid formation, like all amyloidogenesis involves homotypic interactions that result in aggregation with subsequent toxic oligomer and fibril formation. Intracellular aggregation is suppressed by heterotypic interactions between the amyloidogenic precursors and elements of the chaperone system, allowing refolding or transport in the soluble state to either the secretory pathway or to the cellular degradative machinery. In the vast neuropathologic literature describing AD, a number of molecules have been found co-localized in the Aβ deposits. Similarly in the hundreds of publications utilizing the murine AβPP transgenic mice as AD models, there are reports of many manipulations that enhance or diminish the AD phenotype. We have presented a detailed analysis of the evidence suggesting that wild type TTR, a systemic amyloid precursor, can suppress Aβ aggregation *in vitro *and *in vivo *and ameliorate its pathologic effects in a well-validated transgenic mouse model of human AD. In the AD model literature we noted that reports indicating that wild type forms of other proteins rendered amyloidogenic by autosomal dominant mutations, e.g. Bri2 [215], cystatin C [213,214], gelsolin [217,218,238], and perhaps neuroserpin [239] (mutations result in non-amyloid neuropathologic aggregation), seem to be over-represented as a class. They appear to have the capacity to interact with Aβ and in some instances suppress the AD phenotypes in transgenic mouse models (see Table 1). It is possible that the same structural features that predispose these proteins to undergo the homotypic interactions that result in aggregation when affected by a particular structural change as a consequence of mutation, allow the wild type conformers to interact heterotypically to prevent aggregation of similarly susceptible client proteins, in this case Aβ. Whether the phenomenon represents therapeutically exploitable physiology remains to be seen.

## Competing interests

The authors declare that they have no competing interests.

## Authors' contributions

XL and JNB wrote the manuscript. Both authors read and approved the final manuscript.
